# Complementary roles of murine Na_V_1.7, Na_V_1.8 and Na_V_1.9 in acute itch signalling

**DOI:** 10.1038/s41598-020-59092-2

**Published:** 2020-02-11

**Authors:** Helen Kühn, Leonie Kappes, Katharina Wolf, Lisa Gebhardt, Markus F. Neurath, Peter Reeh, Michael J. M. Fischer, Andreas E. Kremer

**Affiliations:** 1 Department of Medicine 1, Friedrich-Alexander-University Erlangen-Nürnberg, Ulmenweg 18, Erlangen, Germany; 2 Institute of Physiology and Pathophysiology, Friedrich-Alexander-University Erlangen-Nürnberg, Universitätsstrasse 17, Erlangen, Germany; 3 Center for Physiology and Pharmacology, Medical University of Vienna, Schwarzspanierstrasse 17, Vienna, Austria

**Keywords:** Molecular biology, Neuroscience

## Abstract

Acute pruritus occurs in various disorders. Despite severe repercussions on quality of life treatment options remain limited. Voltage-gated sodium channels (Na_V_) are indispensable for transformation and propagation of sensory signals implicating them as drug targets. Here, Na_V_1.7, 1.8 and 1.9 were compared for their contribution to itch by analysing Na_V_-specific knockout mice. Acute pruritus was induced by a comprehensive panel of pruritogens (C48/80, endothelin, 5-HT, chloroquine, histamine, lysophosphatidic acid, trypsin, SLIGRL, β-alanine, BAM8-22), and scratching was assessed using a magnet-based recording technology. We report an unexpected stimulus-dependent diversity in Na_V_ channel-mediated itch signalling. Na_V_1.7^−/−^ showed substantial scratch reduction mainly towards strong pruritogens. Na_V_1.8^−/−^ impaired histamine and 5-HT-induced scratching while Na_V_1.9 was involved in itch signalling towards 5-HT, C48/80 and SLIGRL. Furthermore, similar microfluorimetric calcium responses of sensory neurons and expression of itch-related TRP channels suggest no change in sensory transduction but in action potential transformation and conduction. The cumulative sum of scratching over all pruritogens confirmed a leading role of Na_V_1.7 and indicated an overall contribution of Na_V_1.9. Beside the proposed general role of Na_V_1.7 and 1.9 in itch signalling, scrutiny of time courses suggested Na_V_1.8 to sustain prolonged itching. Therefore, Na_V_1.7 and 1.9 may represent targets in pruritus therapy.

## Introduction

Pruritus, commonly also referred to as itch, can appear as agonizing symptom of dermatological, systemic and psychogenic disorders^1^. Despite its high impact on the quality of life, treatment options remain limited. One obstacle in the development of new drugs arises from the diversity of signalling pathways triggering itch. Aside histamine, which was the first known pruritogen^2,3^, pruritus can be elicited by various histamine-independent pruritogens with diverse chemical structures acting on a wide range of receptors. A major role in histamine-independent itch signalling can be attributed to Mas-related G protein-coupled receptors (MRGPR). This family of G protein-coupled receptors (GPCRs) mediates acute scratch behaviour towards pruritogens such as bovine adrenal medulla 8–22 peptide (BAM8-22)^4^, chloroquine^5^, β-alanine^6^ and the tethered PAR2 ligand SLIGRL^7^. Additionally, pruritogens like 5-hydroxytryptamine (5-HT)^8^, and lysophosphatidic acid (LPA)^9^ signal via activation of their specific receptors.

Pruritogens can activate GPCRs located on nerve endings of unmyelinated primary sensory neurons, resulting in a rise of cytosolic calcium levels both via the inositol phospholipid signalling pathway^10–13^ and via transient receptor potential ion channels (TRP)^14^. Upon membrane depolarization, voltage-gated sodium channels (Na_V_) are opened, triggering the initiation and propagation of action potentials. The function of Na_V_ channels is determined by the pore-forming alpha-subunit of which nine subtypes with distinct functions have been identified^15^. Primary sensory neurons express virtually all subunits^16,17^. However, transcription analysis using single neuron RT-PCR and RNA sequencing have revealed a vast abundance of Na_V_1.7, 1.8 and 1.9 in the slow conducting unmyelinated sensory neurons^18,19^. Complementary physiological studies have shown that these channels modulate pain signalling^20–22^. For their indispensable role in the generation and propagation of action potentials, these Na_V_ subtypes have been suggested as potential drug targets for blunting sensory perceptions. Several subtype-specific Na_V_ inhibitors have lately entered clinical trials for different pain treatments^23–25^.

Additionally, recent studies presented an important role of Na_V_1.7–1.9 in itch signalling. Case studies revealed that gain-of-function mutations in these Na_V_ channels can cause paroxysmal itch in affected patients^26–29^. Corresponding inhibitory studies analysed the role of Na_V_ subtypes in itch signalling. Inhibition of Na_V_1.7 reduced acute scratch behaviour in mice upon histamine and selected non-histaminergic stimuli^19,30–33^. Moreover, Na_V_1.9 knockout mice showed reduced scratch behaviour upon treatment with histamine, chloroquine and BAM8-22^28^. This suggests that Na_V_1.7 and Na_V_1.9 are involved in acute itch signalling. While an isolated analysis of individual Na_V_ channels is sufficient to determine a role of Na_V_ subtypes in acute itch, the complexity of itch signalling with a possible complementing function of Na_V_ channels, as indicated by recent *in vitro* studies^18^, remains elusive. To create a comprehensive picture of the role of Na_V_ channels in acute itch signalling we utilized knockout models for Na_V_1.7, 1.8 and 1.9 and analysed the complementary roles of the Na_V_ channels for diverse itch stimuli. We show here that various pruritogens require different Na_V_ channels to mediate an itch stimulus while neuronal activation is unaltered in primary sensory neurons of the respective knockout animals.

## Results

### Acute itch stimuli signal via different Na_V_ channels

**Figure 1:**
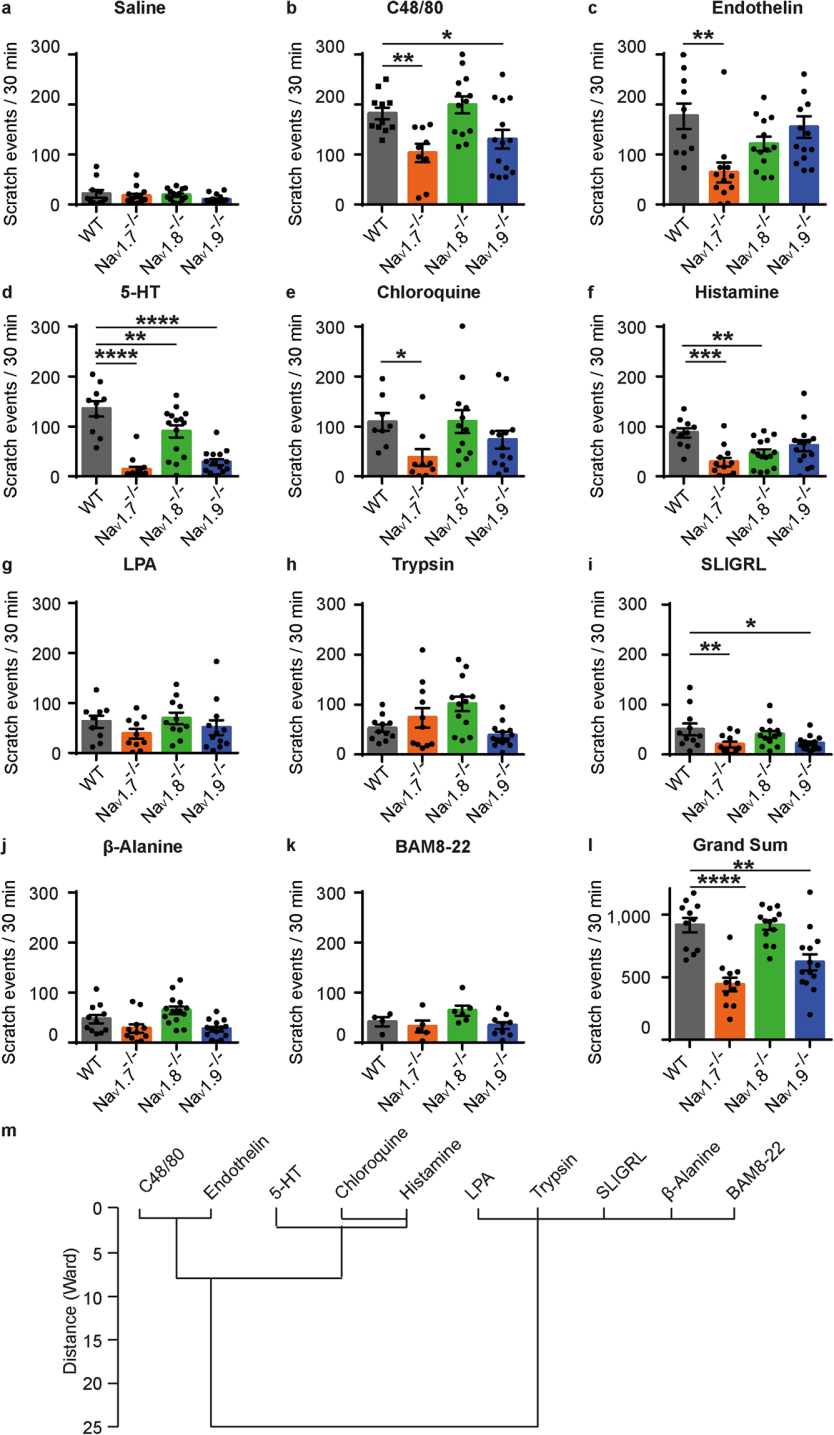
Acute scratch behaviour in Na_V_1.7^−/−^, Na_V_1.8^−/−^, Na_V_1.9^−/−^ and wild type mice upon intradermal injection of pruritogens. (**a–k**) Scratch events within 30 min after intradermal injection (50 µl) of saline, C48/80 (2 g/l), endothelin (1 µM), 5-HT (1 mM), chloroquine (4 mM), histamine (89 mM), LPA (4 mM), trypsin (10 U/µl), SLIGRL (2 mM), ß-alanine (224 mM) and BAM8-22 (1 mM). Pruritogens are displayed in order of potency, sorted according to the mean scratch behaviour induced in wild type mice. (**l**) Grand sum of the scratch behaviour across all pruritogens, n = 4–15, error bars: s.e.m, *P < 0.05, **P < 0.01, ***P < 0.001, ****P < 0.0001 (**m**) Hierarchical cluster analysis of the average scratch behaviour induced by each pruritogen in wild type mice using Ward’s method.

Scratch behaviour upon diverse acute itch stimuli was assessed in Na_V_1.7^−/−^, Na_V_1.8^−/−^, Na_V_1.9^−/−^ knockout mice and congenic wild type animals in order to explore which Na_V_ channels are required for itch signalling. All pruritogens were applied intradermally in the nape at concentrations which were, according to several publications, described to induce substantial itch (Fig. 1a–k). Scratch bouts were quantified for 30 min using an observer-independent, automated recording system. Scratching, assessed by a two-way ANOVA, showed a significant interaction of the between-subject factor ‘genotype’ and the within-subject factors ‘pruritogen’ (F_(30,430)_ = 4.36, P < 0.0001) but revealed no differences for the factor ‘sex’ (F_(1, 528)_ = 1.48, p = 0.22, including no interaction between ‘sex’ and ‘genotype’ p = 0.55, Supplementary Fig. S1 online). As the pruritogenic potential varied among the different pruritogens, we assessed the similarities in the induced scratch response in wild type mice using hierarchical cluster analysis with the Ward’s method (Fig. 1m). The average scratch responses in wild type mice clustered into two groups. Thereby, the first cluster comprised pruritogens with strong and medium scratch responses (88–182 scratch events/30 min), namely C48/80, endothelin, 5-HT, chloroquine and histamine (Fig. 1b–f). The second group contained the pruritogens LPA, trypsin, SLIGRL, β-alanine and BAM8-22 inducing weaker scratching in mice (47–62 scratch events/30 min, Fig. 1g–k). All potent pruritogens from the first cluster showed a dependency on the expression of at least one of the investigated Na_V_ channels (Fig. 1b–f). Na_V_1.7 knockout mice had a significantly reduced scratch behaviour upon C48/80 (P = 0.001), endothelin (P = 0.002), 5-HT (P < 0.001), chloroquine (P = 0.028) and histamine (P < 0.001, prespecified contrasts against wild type animals, Fig. 1b–f). Na_V_1.8^−/−^ exhibited a significantly reduced scratching upon 5-HT (P = 0.009) and histamine (P = 0.006), while Na_V_1.9 knockout reduced the scratching upon C48/80 (P = 0.017) and 5-HT (P < 0.001).

Among the weaker pruritogens, SLIGRL-induced scratching was reduced upon Na_V_1.7 and Na_V_1.9 deletion (P = 0.005 and 0.033, prespecified contrasts against wild type animals). In contrast, the scratch responses upon the other weaker pruritogens were not significantly affected by the deletion of individual Na_V_ channels (P = 0.166–0.940, prespecified contrasts against wild type animals), albeit there was a trend towards reduced scratching activity in both Na_V_1.7^−/−^ and Na_V_1.9^−/−^ mice. Taken together, the grand sum of scratch responses over all pruritogens showed a 51.8% reduction of scratching in Na_V_1.7^−/−^ and 32.4% in Na_V_1.9^−/−^ but not in  Na_V_1.8^−/−^ (post-hoc test for ‘genotype’; P < 0.001, P = 0.006, P = 1.0, Fig. 1l) independent of the variance of individual pruritogens. Of note, all stimuli which were significantly modulated by Na_V_1.8 or Na_V_1.9 also exhibited a significant dependency on the expression of Na_V_1.7.

### Deletion of Na_V_ channels affects different phases of acute scratching

**Figure 2:**
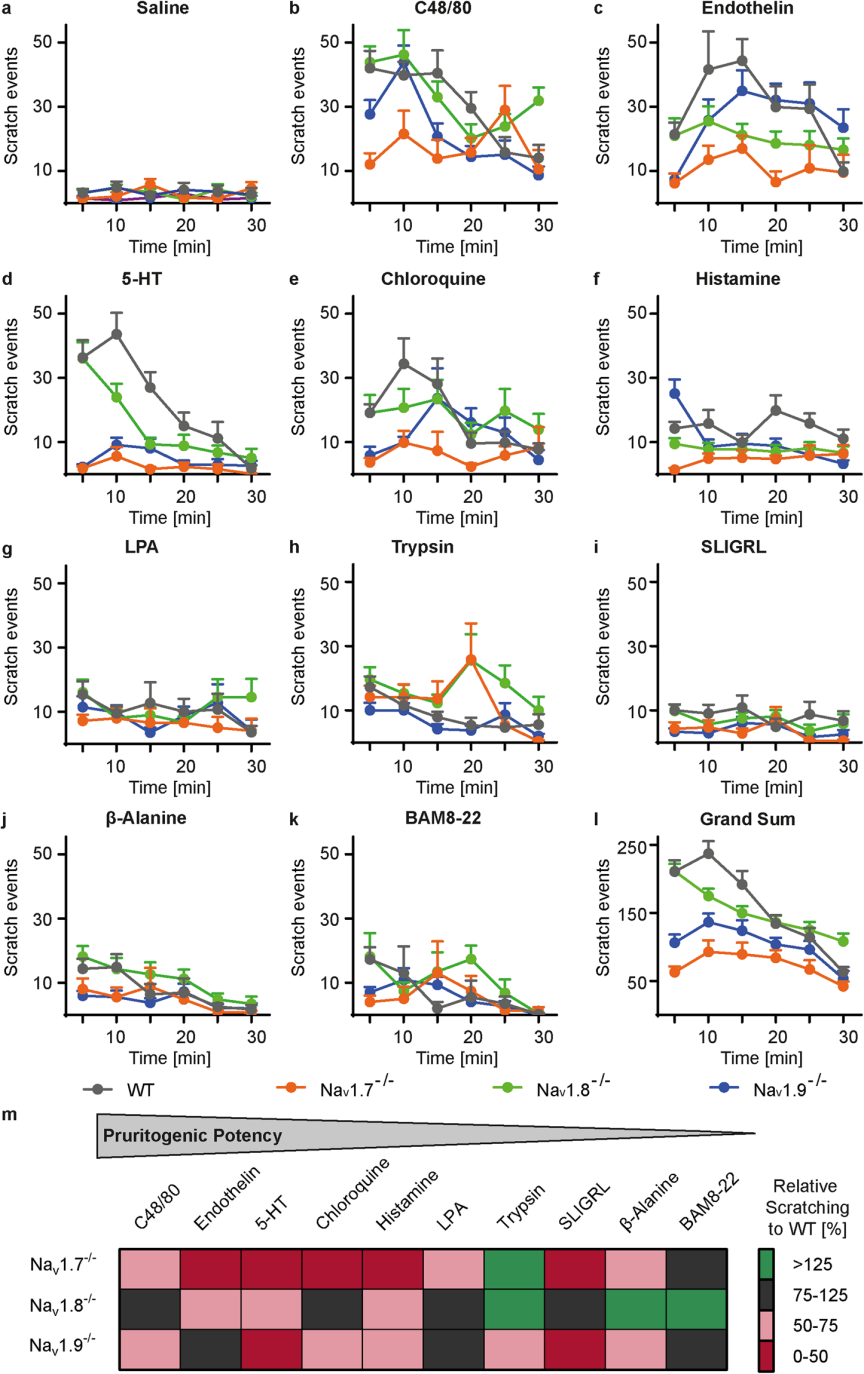
Na_V_1.7^−/−^, Na_V_1.8^−/−^ and Na_V_1.9^−/−^ mice exhibit different time-resolved scratch patterns. (**a–k**) Time course of scratch behaviour after intradermal injection (50 µl) of saline, C48/80 (2 g/l), endothelin (1 µM), 5-HT (1 mM), chloroquine (4 mM), histamine (89 mM), LPA (4 mM), trypsin (10 U/µl), SLIGRL (2 mM), ß-alanine (224 mM) and BAM8-22 (1 mM) (**l**) Grand sum of the scratch behaviour across all pruritogens; error bars: s.e.m (**m**) Heat map of the relative scratch behaviour in Na_V_1.7^−/−^, Na_V_1.8^−/−^, Na_V_1.9^−/−^ mice compared to wild type mice. Pruritogens are displayed in order of potency, sorted according to the mean scratch behaviour induced in wild type mice.

Time courses were scrutinized to assess which phases of scratching were affected by deletion of individual Na_V_ channels. Exploratory analysis of the time courses of scratching for the different stimuli showed different scratch patterns in the respective genotypes (Fig. 2a–l). The time-resolved grand sum of scratching exhibited in wild type mice an increase to a maximum at ten minutes after injection and then a linear decrease with a time constant of about 27 min (Fig. 2l). Na_V_1.7 and Na_V_1.9 knockouts showed the same pattern of time-related behaviour but on a much lower level of event counts (two-way ANOVA genotype x period P < 0.0001). In contrast, Na_V_1.8^−/−^ mice began at 5 min with the same high number of scratch events as wild types but then declined monotonously without a further peak. They clearly showed no scratch peak after injection of endothelin, 5-HT and histamine (Fig. 2c,d,f) resulting in reduced total scratching compared to wild type mice (Fig. 2m).

### Pharmacological inhibition of Na_V_1.7 and Na_V_1.8 reduces acute scratching upon histamine and endothelin

**Figure 3:**
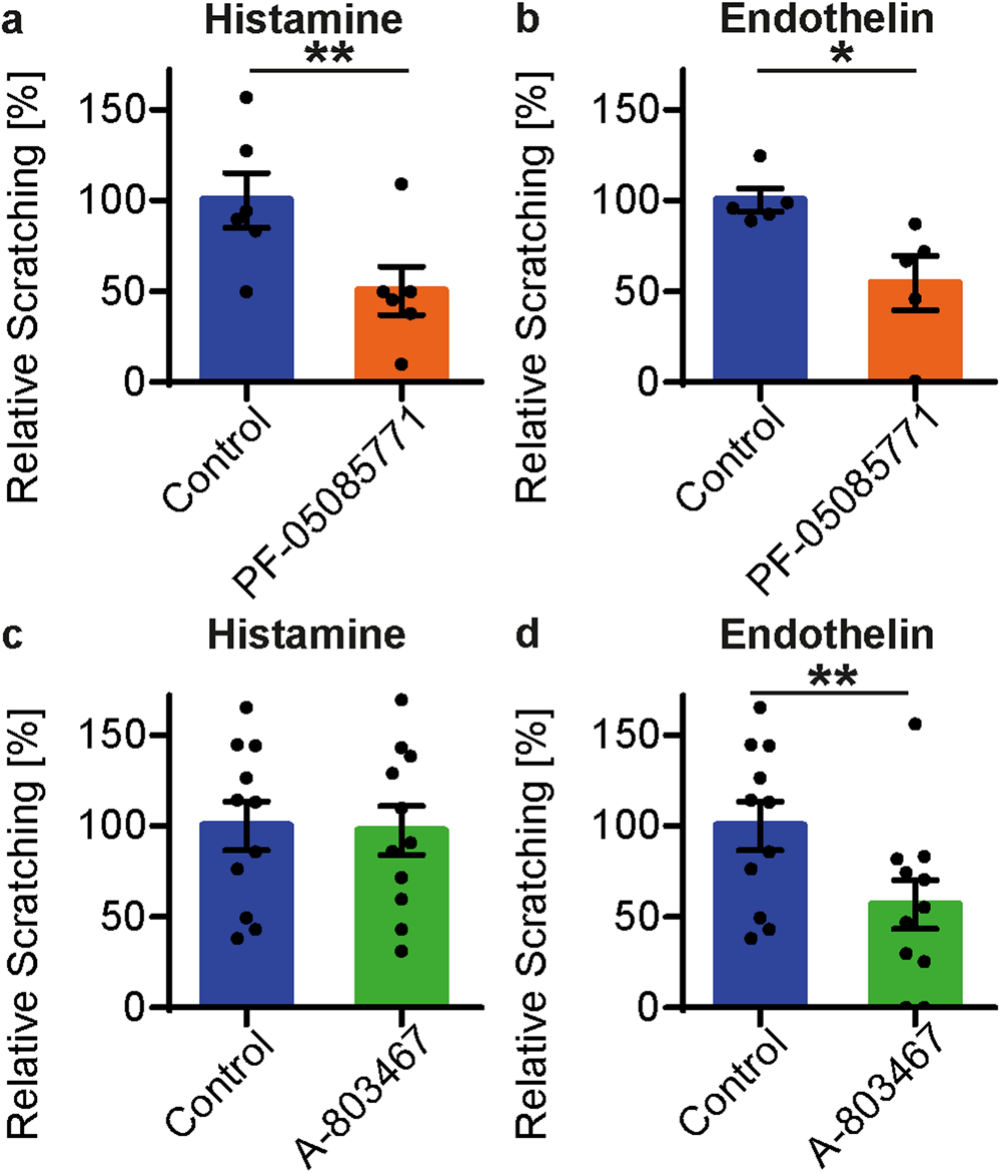
Reduction of histamine- and endothelin-induced scratching through Na_V_1.7 and Na_V_1.8 inhibition. Scratch behaviour upon intradermal injection of histamine (89 mM) and endothelin (1 µM) following inhibitor treatment relative to control response upon saline injection. (**a,b**) Pre-treatment with Na_V_1.7 inhibitor PF-05089771 (i.p., 90 µg) 3 h prior to acute itch induction. (**c,d**) Pre-treatment with Na_V_1.8 inhibitor A-803467 (i.p., 1.75 mg) 1.5 h before itch assessment. n = 5–12, error bars: s.e.m, *P < 0.05, **P < 0.01.

To further corroborate the role of Na_V_ channels in acute itch signalling observed in genetic deletion models, we assessed the potential of Na_V_1.7 inhibitor PF-05089771^34,35^ and Na_V_1.8 inhibitor A-803467^36^ to alleviate acute scratching upon histamine and endothelin. As there is no specific Na_V_1.9 inhibitor commercially available, the effects of pharmacological Na_v_1.9 inhibition could not be assessed. Inhibition of Na_V_1.7 by intraperitoneal pre-treatment of mice with PF-05089771 led to a reduced scratching upon histamine by 49.8% (paired t-test, P < 0.001) and upon endothelin by 45.6% (paired t-test, P = 0.031, Fig. 3a,b). Pharmacological inhibition of Na_V_1.8 through intraperitoneal application of A-803467 prior to the intradermal pruritogen injection reduced scratch behaviour upon endothelin by 43.3% (paired t-test, P* = *0.003) while the histamine response remained unaffected (paired t-test, P = 0.77, Fig. 3c,d).

### Activation of signalling pathways upstream of Na_V_ channels is equivalent in knockout and wild type mice

**Figure 4:**
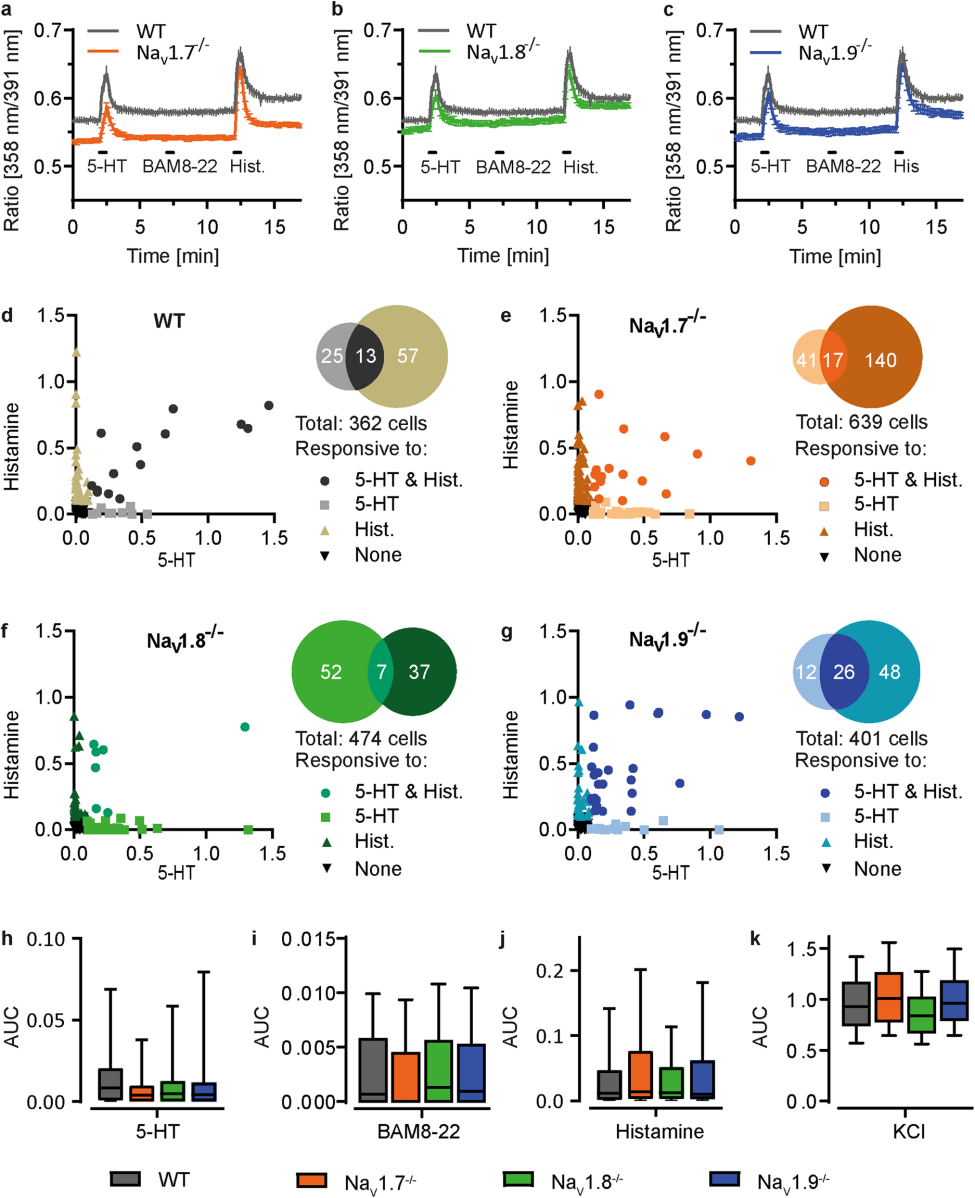
Dorsal root ganglion neurons from Na_V_1.7^−/−^, Na_V_1.8^−/−^, Na_V_1.9^−/−^ and wild type mice show similar calcium responses upon stimulation with 5-HT, BAM8-22 and histamine. (**a**–**c**) Average cytosolic calcium transients in dissociated, 1-day cultured dorsal root ganglion neurons upon stimulation with 5-HT (1.5 µM), BAM8-22 (2 µM), histamine (150 µM) and KCl (60 nM) as positive control. Bars indicate application periods. (**d**–**g**) Scatterplots of the ratio increases of individual neurons of Na_V_1.7^−/−^, Na_V_1.8^−/−^, Na_V_1.9^−/−^ and wild type mice responding to histamine and 5-HT. Venn diagrams illustrate the overlap between cells positive for either or both stimuli (**h**–**k**) Box plots of the corresponding area under the curve (AUC)  for the first minute of application. Boxes display median and 25–75 percentiles; whiskers represent 10 and 90 percentiles.

To verify that a reduction of acute scratch behaviour in knockout mice is related to a deletion of the respective Na_V_ channel and not caused by upstream effects, we compared the potential of the pruritogens to activate dissociated dorsal root ganglia neurons (DRGs). Cultured sensory neurons of both wild type and knockout mice exhibited similar increases in cytosolic calcium upon addition of histamine, BAM8-22 and 5-HT (Fig. 4a–c). Furthermore, a similar percentage of neurons from wild type and knockout mice responded to histamine and serotonin, respectively (one-way ANOVA, F_(3, 11)_ = 5.04 & 0.33; Na_V_1.7^−/−^: P = 0.14 & 0.99; Na_V_1.8^−/−^: P = 0.33 & 0.97; Na_V_1.9^−/−^: P = 0.93 & 0.69). The corresponding pairwise scatterplots and respective Venn diagrams showed a similar distribution of neurons reacting to 5-HT and/or histamine in wild type and knockout animals (Fig. 4d–g). Accordingly, the histamine-induced transient cytosolic calcium increase displayed in the area under the curve was comparable in knockout and wild type animals (Fig. 4h–k).

**Figure 5:**
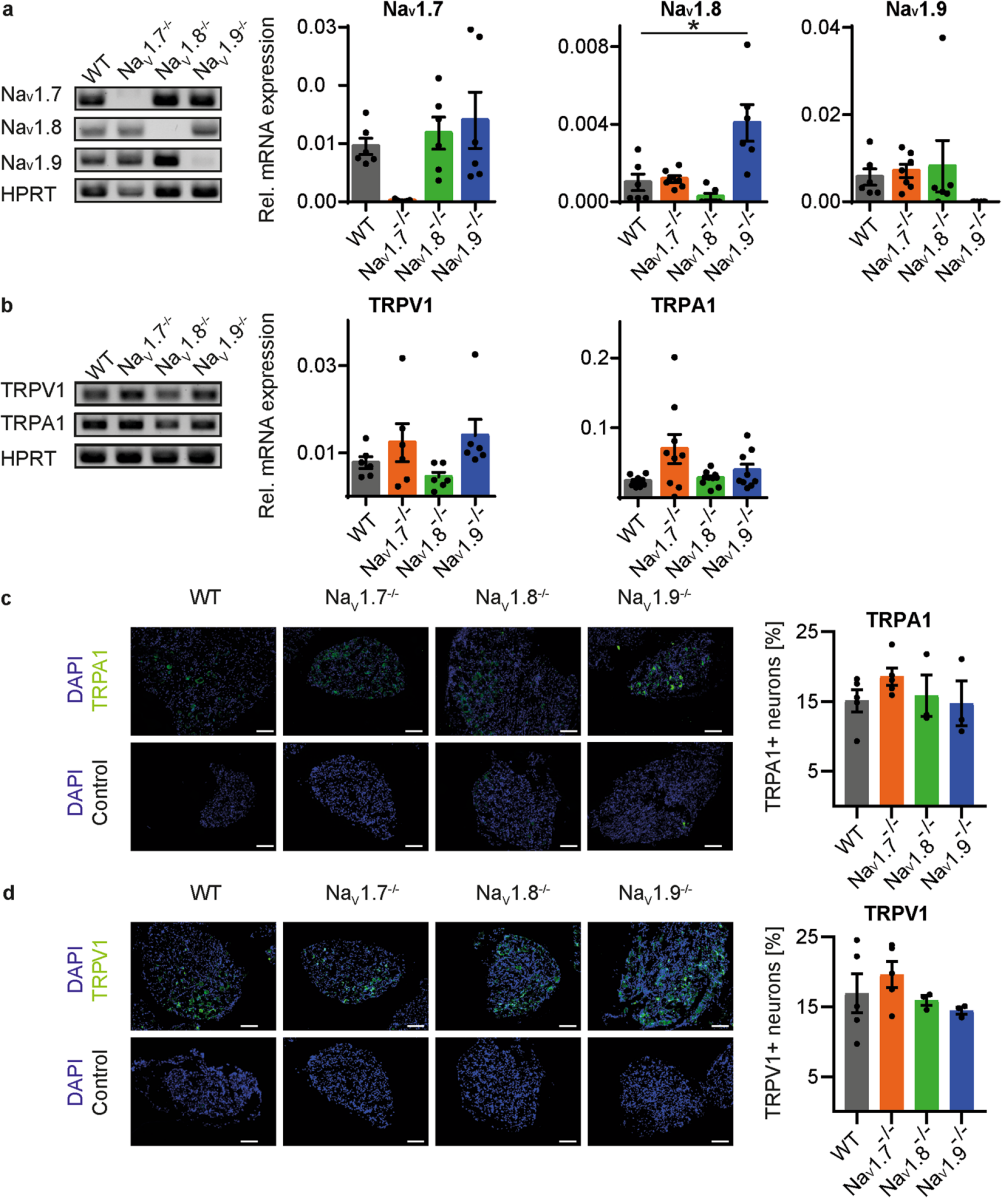
Expression of Na_V_ and TRP channels in dorsal root ganglion neurons of Na_V_1.7^−/−^, Na_V_1.8^−/−^, Na_V_1.9^−/−^ and wild type mice. (**a,b**) Relative mRNA expression compared to HPRT and respective agarose gels of amplified PCR products for Na_V_, TRPA1 and TRPV1 channels. error bars: s.e.m, *P < 0.05 (**c**,**d**) Representative and quantitative immunohistochemistry of TRPA1 and TRPV1 expression, n = 3–5 (average from 4–7 slices per animal), error bars: s.e.m, scale bar: 100 µM.

Furthermore, we assessed the expression of the investigated Na_V_ channels on mRNA level. Na_V_1.7^−/−^ and Na_V_1.8^−/−^ revealed no compensatory up- or downregulation of the other Na_V_ channels on mRNA level, while Na_V_1.9^−/−^ mice showed a 4-fold upregulation of Na_V_1.8 (U-Test, P = 0.036; Fig. 5a). As itch signalling pathways can be modulated by transient receptor potential ion channels (TRP), we further investigated the expression of TRPA1 and TRPV1 on mRNA and protein level. In agreement with the cellular responses detected by measurement of cytosolic free calcium levels, expression of TRPA1 and TRPV1 was comparable in knockout and wild type animals (Kruskal-Wallis Test, Fig. 5b–d). Corresponding immunofluorescence showed no difference in expression or localization of TRPA1 and TPRV1 in knockout- and wild type animals (Fig. 5c,d).

## Discussion

Pruritus represents a clinical burden which can occur in various systemic diseases, under dermatological conditions and as adverse effect of countless therapies^1,37^. To date treatment options remain limited and the diversity of pruriceptors and signalling pathways involved as well as their redundancy represent major challenges in the development of novel anti-pruritic treatment strategies. As Na_V_ channels are comparably few but indispensable for the transformation and propagation of sensory signals, they represent common key players for the signalling of pruritus independent of its origin and have therefore been suggested as drug targets. To widen our knowledge about the role of Na_V_ channels in acute itch signalling, we compared the involvement of Na_V_1.7, Na_V_1.8 and Na_V_1.9, performing behavioural studies with a comprehensive set of pruritogens in the respective knockout mice. The results revealed an unexpected diversity of the Na_V_-mediated itch signalling depending on the acute stimulus applied. Deletion of either Na_V_1.7, Na_V_1.8 or Na_V_1.9 had an impact on acute itch signalling albeit to different extents. Na_V_1.7 deletion blunted most acute itch stimuli and Na_V_1.9 had a robust overall effect on itch signalling, while Na_V_1.8 deletion reduced scratch behaviour only towards certain strong stimuli and resulted in a different time course of scratching. Thereby, equal microfluorimetric calcium responses of sensory neurons in the regarded genotypes and a similar expression of itch-related TRP channels, suggested no change in sensory transduction but in action potential transformation and conduction. Differences in scratch behaviour can therefore be attributed to Na_V_ deletion and not to changes upstream of Na_V_.

Deletion of Na_V_1.7 affected the signalling of all tested pruritogens – ranging from moderate to strong reduction of scratch behaviour. This goes in line with previous studies showing a reduction of histamine-, chloroquine- and C48/80-induced itch^30,31,38^. While those studies confirmed the role of Na_V_1.7 for individual pruritogens, our results strongly suggest Na_V_1.7 to be a key mediator of acute itch signalling independent of the stimulus applied. Na_V_1.7 is characterized by a low threshold and rapid activation^39^. It has therefore been suggested to trigger action potentials and contribute to determining nociceptor excitability^40^. This hypothesis has been supported by observations in patients with inherited erythromelalgia caused by different point mutations in SCN9A. Mutations in the gene for Na_V_1.7 lead to a hyperpolarizing shift of the voltage-dependent activation of the channel^41–43^. In contrast, loss-of-function mutations in the same gene lead to the Congenital Indifference to Pain (CIP) syndrome which includes the lack of pruritus^44^. The substantial reduction of scratching in Na_V_1.7^−/−^ and the failure of knockout mice to reach scratch peaks in comparison to the congenic wild type mice, indicate that this channel is most relevant for the transformation of itch stimuli in mice, though not indispensable. Of note, the used Na_V_1.7^−/−^ mouse strain underlies an Advillin-Cre based conditional deletion of Na_V_1.7 in sensory neurons. Therefore, a marginal neuronal Na_V_1.7 expression cannot be ruled out and a germline knockout out model may show an even more prominent role of Na_V_1.7 in acute itch signalling as suggested by Gingras *et al*.^45^. However, as Na_V_1.7 mRNA expression in DRGs was undetectable, the risk of underestimating the Na_V_1.7 expression and its influence on itch behaviour appears minimal.

Despite the high expression of Na_V_1.8 in sensory neurons activated by itch and pain^18^ as well as the occurrence of chronic pruritus in patients with a gain-of-function mutation^27^, the role of Na_V_1.8 in itch signalling remains poorly understood. Here, we show that Na_V_1.8 modulates scratch behaviour induced, in particular, by the strong pruritogens 5-HT and histamine. Albeit there was not a uniform change in the responses to different pruritogens in Na_V_1.8^−/−^, the grand sum of scratch events across all applied pruritogens (Fig. 2l) suggests that the behavioural activity declines from an initial maximum without the normal exaggeration around 10 min after injection. This may be due to the particular Na_V_1.8 capacity of “fast re-priming”, i. e. fast recovery from voltage-dependent inactivation (brief refractory period). This allows high-frequency repetitive firing of action potentials^46–48^ and thus continuation of high scratch levels. Knockout animals may lose this capacity which may shorten the scratching periods upon strong itch stimuli. Both deletion of Na_V_1.8 and its pharmacological inhibition reduced endothelin-induced scratch behaviour in mice. Knockout animals showed an average reduction of 31% compared to wild type mice which is in line with 46% decrease during pharmacological inhibition. Although reduction of scratching in Na_V_1.8^−/−^ failed to reach significance (P = 0.063), effects of pharmacological inhibition strongly suggest biological relevance and involvement of Na_V_1.8 in endothelin-induced scratching. Surprisingly, deletion of Na_V_1.8 resulted in a significantly reduced scratching induced by histamine while pharmacological inhibition of Na_V_1.8 failed to decrease scratch activity. Jurcakova *et al*. have recently shown that Na_V_1.8 inhibition alone does not affect firing response of murine C-fibers upon chloroquine treatment, and dual inhibition of Na_V_1.7 and 1.8 is needed to abolish this firing^18^. The sufficiency of sole Na_V_1.8 inhibition to reduce acute itch signalling upon endothelin but not histamine, may indicate a different weight of Na_V_ channel participation during acute itch signalling depending on the distinct stimulus. This may finally lead to differential effects of single Na_V_ inhibitors.

The results further demonstrate that Na_V_1.9 deletion results in a substantial overall reduction of acute scratching in the sum of all applied pruritogens and in particular upon intradermal injection of C48/80, 5-HT and SLIGRL. As in Na_V_1.7^−/−^, the reduction of scratch responses was most prominent during the first 15 min of observation. Recent studies showed that Na_V_1.9 knockout mice exhibit a significantly elevated threshold to mechanical and heat stimuli and a reduced (electrical) excitability of skin nociceptors^49^. Taken together with our behavioural data, we suggest that Na_V_1.9 modulates the transformation of acute itch stimuli, lowering the threshold to action potential generation. The effects observed in Na_V_1.9^−/−^ are less prominent than observations recently made by Salvatierra *et al*., who showed a reduction of scratch behaviour in Na_V_1.9 knockout mice upon histamine, chloroquine and BAM8-22 when applying the substances subcutaneously^28^. While the nerve endings of primary sensory neurons largely terminate in the epidermis, other cellular players of itch signalling such as mast cells are distributed in the corium^50^ which potentially modulate neuronal activation when applying pruritogens subcutaneously compared to an intradermal application.

Utilization of knockout models enables the directed examination of channel characteristics and investigates a link between behavioural phenotypes and the expression of the regarded molecule of interest. However, due to the general nature of knockout models, it cannot be excluded that further molecules up- and downstream of the respective Na_V_ channels are affected by the genetic modification and may influence the itch behaviour investigated here. Furthermore, knockout of Na_V_1.8 has been shown to induce upregulation of Na_V_1.7^20,51,52^ while no indications of compensatory effects in Na_V_1.7^−/−^ and Na_V_1.9^−/−^ have been reported^53,54^. Although, compensation could affect behavioural phenotypes of the knockout mice, an upregulation of Na_V_1.7 in Na_V_1.8^−/−^ cannot explain the phenotype observed here. Na_V_1.8^−/−^, lacking the fast re-priming sodium channel, exhibit a normal onset of scratching compared to wild type mice but fail to reach scratch peaks as detected in the grand sum over all pruritogens. In contrast, the tetrodotoxin-sensitive Na_V_1.7 has a slow recovery from fast voltage-dependent inactivation^55,56^ and can therefore not trigger high frequency spiking which occurs with strong pruritogens after injection. Consequently, the observed behavioural abnormalities of Na_V_1.8^−/−^ can be attributed to the deletion of Na_V_1.8 and are not a result of Na_V_1.7 upregulation.

In agreement with previous studies, we show that a deletion or inhibition of one Na_V_ channel is not sufficient to fully abolish scratching in mice^28,30–33^. The contribution of several Na_V_ channels to acute itch signalling of different stimuli, shown here, supports the suggestion that a simultaneous inhibition of different Na_V_ channels is required to reach full abolishment. This is in line with recent studies on *ex vivo* DRG-nerve-skin preparations reporting that tetrodotoxin suppressed firing in 75% of the fibers, Na_V_1.7 selective blockage in 40% of itch C-fibers, but the combination of Na_V_1.7 and Na_V_1.8 inhibition resulted in full abolishment of action potential discharge^18^. Contrary, in human case studies a loss-of-function mutation of Na_V_1.7 resulted in absence of itch perception^44^. As Na_V_1.7 deletion has been shown to cause an increased efficiency of antinociception via μ-opioid receptors^57^, which are involved in itch signalling^58^, it may be possible that μ-opioid signalling effects itch sensitivity in the knockout mice. Furthermore, thin myelinated A-fibers, which express Na_V_1.6 as action potential generator in their nodes of Ranvier, have been shown to contribute to cowage- and histamine-induced itch in primates and humans^59^ and could maintain acute itch signalling to some extent.

In summary, our results demonstrate an involvement of Na_V_1.7, 1.8 and 1.9 in acute signalling. Scrutiny of the scratch pattern in knockout mice revealed a role of Na_V_ in different phases of acute scratching. According to our behavioural data, Na_V_1.7 and 1.9 generally participate in itch signalling, while Na_V_1.8 sustains prolonged itching. Unravelling these molecular mechanisms of itch signalling in the primary sensory neurons will have a major impact on the development of new therapies. In case of acute pruritus, we suggest that Na_V_1.7 and 1.9 may provide targets in pruritus therapy.

## Methods

### Animals

Na_V_1.7^−/−^ mice exhibited an Advillin promoter-dependent deletion of exon 14 and 15 of the SCN9A gene, preventing functional expression of Na_V_1.7 in dorsal root ganglia (DRG) and trigeminal ganglia neurons^60^^.^ Na_V_1.8^−/−^ and Na_V_1.9^−/−^ were generated by deletion of exon 4–5 of SCN10A and SCN11A, respectively, as described before^51,54^. Knockout was confirmed by genotyping as described earlier^51,54,60^. Animals from both sexes with an age from 11 to 19 weeks, bred in house, were used for experiments. Mice were housed in a regulated 12 h day-night cycle with water and nutrition ad libitum. As the knockout strains are backcrossed to C57BL/6 J every 3–4 generations, congenic; age- and sex-matched C57BL/6 J mice were used as controls. Animals were killed in a rising CO_2_ atmosphere and by cervical dislocation. The present study was performed in accordance institutional, national and international guidelines and regulations. All experiments were approved by the institutional animal care (Sachgebiet Tierschutz, Friedrich-Alexander University, Erlangen, Germany) and the district government (55.2.2-2532-2-642-11; Regierung Unterfranken, Würzburg, Germany).

### Behavioural itch assay

The behavioural testing to asses scratch behaviour was conducted as described previously^9^. Briefly, small Teflon-coated magnets were implanted into both hind paws of the experimental animals one week prior to behavioural experiments. The mice were given 60 min to acclimatize to the individual measurement cages before intradermal injection of 50 µl saline or the respective pruritogens into the nape using a 30 G fine dosage syringe (Braun, Kronberg im Taunus, Germany). The mice were injected on 6 consecutive days. After injection of saline on day 1 5-HT (1 mM, Biomol, Hamburg, Germany), histamine (89 mM, Carl Roth, Karlsruhe, Germany), endothelin (1 µM, Enzo, Farmingdale, NY, USA), chloroquine (4 mM, Sigma Aldrich) and LPA 18:1 (4 mM, Avanti Lipids, Alabaster, AL, USA) were applied. After 3 days of rest, consecutive injections of β-alanine (224 mM, Sigma Aldrich, St. Louis), C48/80 (2 g/l, Sigma Aldrich, St. Louis, MO, USA), SLIGRL (2 mM, Biomatik, Cambridge, ON, Canada), trypsin (10 U/µl, Sigma Aldrich) and BAM8-22 (1 mM, Genemed Synthesis Inc., San Antonio, TX, USA) were performed. Power analysis was conducted beforehand to estimate the required animal numbers using G*Power software (Version 3.1.9.2)^61^. All genotypes underwent the same application procedures. A substance-independent sensitization of the animals due to consecutive injections was excluded by unchanged scratch responses upon saline injection before and after other itch-inducing stimuli. Furthermore, application order was potency adapted varying between strong and weak pruritogens. The repeated administration did not cause any visible local damage to the skin, which was checked each day before intradermal administration of the substances. The protocol and experimental design was known to the experimenter since the automated scratch recording guaranteed unbiased scratch assessment. Immediately after injection, scratch behaviour was assessed for 30 min. Scratches were automatically detected as the movement of the implanted magnets induced electric currents in two coils around the cages, recorded using oscillography. Recordings were controlled by SiMon (V2.0, Academic Medical Center, University of Amsterdam, the Netherlands) and analysed offline by Scratch Analysis (V1.13, Academic Medical Center, University of Amsterdam, the Netherlands). Movements were classified as scratching based on their frequency (10–20 Hz), the amplitude of the signals (above 300 mV) and a minimum of 4 repetitions. For this methodical approach a positive prediction value of 95% was shown before^9^.

For pharmacological inhibition of Na_V_1.7, 90 µg of PF-05089771 (Sigma Aldrich) were dissolved in extracellular solution (145 mM NaCl, 4.96 mM KCl, 1.62 mM CaCl_2_, 0.98 mM MgCl_2_, 10 mM Glucose, 10 mM Hepes, pH: 7.4) supplemented with 1% dimethyl sulfoxide (DMSO, Carl Roth) and applied intraperitoneally 3 h prior acute itch measurement. Na_V_1.8 was inhibited using 1.75 mg of A-803467, dissolved in polyethylene glycol 400 (PEG 400, Sigma Aldrich) supplemented with 5% DMSO and applied intraperitoneally 1.5 h before scratch measurement. The control groups underwent the same experimental procedures but received intraperitoneal injection of the respective solvent.

### Immunofluorescence

For DRG isolation, the spinal column of sacrificed mice was removed and incubated for 2 h in 4% paraformaldehyde (PFA). Subsequently, DRGs were isolated and incubated for another 30 min in 4% PFA before being transferred to phosphate buffered saline (PBS) followed by 20% sucrose solution each for 24 h at 4 °C. Then, the tissue was embedded in Tissue-Tek O.C.T. Compound (Sakura Alphen aan den Rijn, The Netherlands), sliced to 14 µM sections using a Leica CM3050S cryostat (Leica, Wetzlar, Germany) and embedded on microscope slides coated with Poly-L-Lysine. After thawing the microscope slides for 1 h at room temperature (RT) and washing the tissue in PBS, unspecific binding sites were blocked in PBS supplemented with 0.5% Triton X-100 (Sigma Aldrich), 1% bovine serum albumin (Sigma Aldrich) and 5% donkey serum (Dianova, Hamburg, Germany) for 1 h at RT. After washing the tissue with PBS, the primary antibody for TRPV1 (Neuromics, Edina, MN, USA; RA14113, polyclonal rabbit, 1:1000) or TRPA1 (abcam, Cambridge, UK; ab62053, polyclonal rabbit, 1:1000) was diluted in blocking solution and incubated overnight at 4 °C. Subsequently, the tissue sections were washed in PBS before incubating the appropriate secondary antibody (donkey-anti-rabbit-Alexa 555, Molecular Probe, Eugene, OR, USA, A-31572, 1:1000) for 1 h at RT. After repeated washing with PBS, the tissue sections were mounted with Roti-Mount FluorCare DAPI (Carl Roth, Karlsruhe, Germany) and staining was visualized using a Leica DM 6000B upright and a Leica TCS SP8 confocal microscope operated by Leica application suite. Images were acquired with a 0.5 NA 20x objective lens and the confocal pinhole was set to 1 Airy unit. The Alexa555-labeled secondary antibody was detected with the 488 nm laser line at emission wavelengths from 493 nm–739 nm. DAPI was measured with the 405 nm laser line at emission wavelengths from 410 nm–493 nm.

### Fluorometric measurement of cytosolic free calcium levels

Isolated DRGs from Na_V_1.7^−/−^, Na_V_1.8^−/−^, Na_V_1.9^−/−^ as well as wild type mice were digested in extracellular solution supplemented with 0.5% streptomyces proteinase and 1% clostridium collagenase (Sigma Aldrich, St. Louis, MO, USA) at 37 °C and 5% CO_2_ for 30 min. Thereafter, the dissociated cells were plated on cover slides precoated with Poly-D-Lysine, and cultivated for 14–20 h in TNB100 Medium supplemented with TNB 100 protein-lipid complex (Biochrom, Berlin, Germany), penicillin and streptomycin (100 U/ml each, Life Technologies, Carlsbad, CA, USA) and nerve growth factor (mouse NGF 2.5 S, 100 ng/ml; Alomone Labs, Tel Aviv, Israel). For calcium imaging, the cells were loaded with the calcium sensitive dye Fura2-AM (3 µM, Biotium, Fremont, CA, USA) diluted in extracellular solution supplemented with 0.02% pluronic F-127 (ThermoFisher Scientific, Waltham, MA or Biotrend, Cologne, Germany). Loading was performed for 30 min at 37 °C and 5% CO_2_. After washing, cover slides were placed on an inverted microscope and samples were excited at 358 und 391 nm with a Polychrome V monochromator (Till Photonics, Graefelfing, Germany) at 1 Hz. A gravity driven and software-controlled common outlet perfusion system^62^ allowed the continuous superfusion of the cells at a rate of 0.5 ml/min. A peltier-cooled slow-scan CCD camera collected the fluorescence emission above 440 nm. Using this perfusion system cells were treated with 1.5 µM 5-HT, 2 µM BAM8-22, 150 µM histamine for 30 s and 60 mM potassium chloride (KCl) for 20 s. All substances were dissolved in extracellular solution. TillVision software (ThermoFisher Scientific) was used to control the experiments, to analyse the data and to calculate the fluorescence ratio (358/391 nm) for all regions of interest after background subtraction. Cells with an increase of fluorescence intensity of minimum 0.1 were considered responding. Negative ratios were set to 0. The area under the curve (AUC) of the ratio within one minute after start of application was calculated. All protocols contained a final application of KCl to discard non-vital and non-neuronal cells.

### Quantitative real-time PCR

Immediately upon isolation, DRGs were transferred to TRIzol (Thermo Fisher Scientific) and homogenized using a TissueLyser (Qiagen, Hilden, Germany). Subsequently, RNA was isolated according to manufacturer’s protocol (TriZOL, Thermo Fisher Scientific). The quality of RNA was assessed using a Nanodrop ND1000 Spectrophotometer (Thermo Fisher Scientific). Complementary DNA was synthesized from 500 ng to 1 µg RNA using oligo-dT primer and SCRIPT cDNA Synthesis Kit (Jena Bioscience, Jena, Germany). Quantitative real-time PCR was performed for 40 cycles at an annealing temperature of 60 °C using SensiFast Sybr No-ROX Kit (Bioline, London, UK) in a CFX Connect qPCR System (Bio-Rad Laboratories, Hercules, CA, USA). Primer sequences are listed in supplementary table S1. The detected quantification cycles (C_q_) were normalized to C_q_ values of the housekeeping gene hypoxanthin-guanin-phosphoribosyltransferase (HPRT) using the 2^−ΔΔCT^ method^63^. The amplified DNA products were separated by agarose gel electrophoresis on a 2% agarose gel supplemented with Midori Green Advanced (Nippon Genetics Europe, Dueren, Germany) and visualized using a Gel Doc XR + Gel Documentation System (Biorad, Hercules, CA, USA).

### Data analysis

Statistical analyses were carried out using Sigmaplot (Version 12.5, Systat Software Inc., Erkrath, Germany) and GraphPad Prism Version 7 and 8 (GraphPad Software, San Diego, CA, USA). Normal distribution was tested using the Kolmogorov-Smirnov test and Shapiro-Wilk test. Normally distributed data of two groups were compared by t-test. For 3 or more groups and repeated measurements, missing data were imputed by mean substitution, and an ANOVA was conducted followed by prespecified constrasts against wild type animals. In case of normal distribution was not given, two groups were compared by a U-test. *P < 0.05, **P < 0.01, ***P < 0.001, ****P < 0.0001. All data are presented as mean ± s.e.m. Hierarchical cluster analysis of the pruritogens was conducted with the mean scratch values of wild type mice using IBM SPSS Statistics (Version 21, IBM, Armonk, NY, US). Distances were calculated using Ward’s methods and the squared Euclidean distance.

## Supplementary information

Supplementary information.

## Data Availability

The datasets generated during the current study are available from the corresponding author on reasonable request.
