# Supplementary material for: Complementary roles of murine NaV1.7, NaV1.8 and NaV1.9 in acute itch signalling
Source: Sci Rep. 2020 Feb 11;10:2326. doi: 10.1038/s41598-020-59092-2 (PMC7012836; doi:10.1038/s41598-020-59092-2)
Supplement: Supplementary file 1 — Supplementary information. [file 41598_2020_59092_MOESM1_ESM.pdf]

Supplementary Information

Complementary roles of murine $Na_V1.7$, $Na_V1.8$ and $Na_V1.9$ in acute itch signalling

Helen Kühn, Leonie Kappes, Katharina Wolf, Lisa Gebhardt, Markus F. Neurath, Peter Reeh, Michael Fischer, Andreas E. Kremer

Supplementary Figure S1 – No sex differences in the acute scratch behaviour in $Na_V1.7^{-/-}$, $Na_V1.8^{-/-}$, $Na_V1.9^{-/-}$ and wild type mice upon intradermal injection of pruritogens**.** (**a-j**) Data are identical to Figure 1, but separated by sex. Scratch events within 30 min after intradermal injection, concentrations of substances as in Figure 1: C48/80 (2 g/l), endothelin (1 µM), 5-HT (1 mM), chloroquine (4 mM), histamine (89 mM), LPA (4 mM), trypsin (10 U/µl), SLIGRL (2 mM) and ß-alanine (224 mM). Pruritogens are displayed in order of potency, sorted according to the mean scratch behaviour induced in wild type mice. (**k**) Grand sum of the scratch behaviour across all displayed pruritogens. The number of animals is visualized by individual dots in the bar charts. error bars: s.e.m

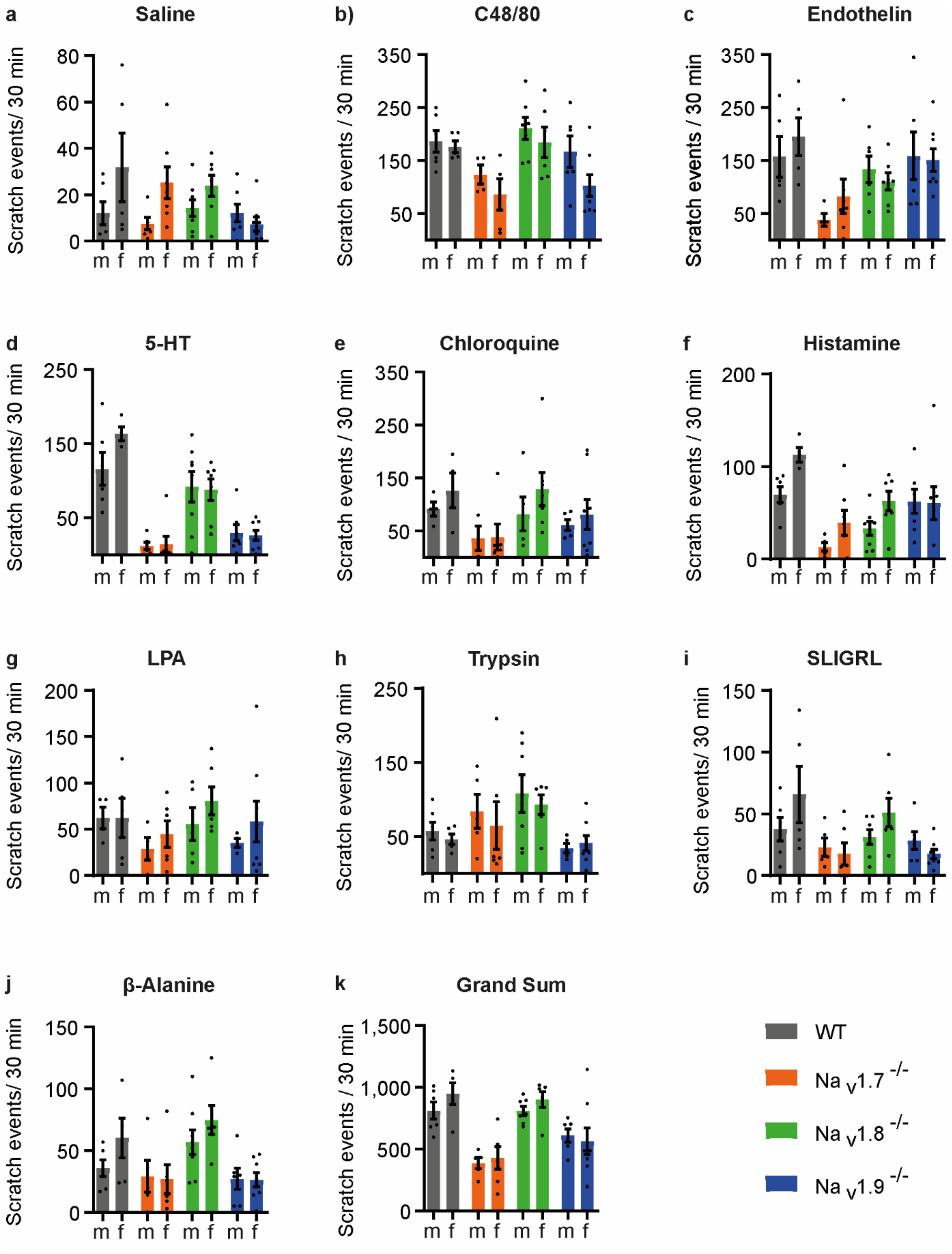

a
Saline
b)
C48/80
c
Endothelin
d
5-HT
e
Chloroquine
f
Histamine
g
LPA
h
Trypsin
i
SLIGRL
j
β-Alanine
k
Grand Sum
Scratch events/ 30 min
Scratch events / 30 min
m f m f m f m f
WT
Na v1.7 -/-
Na v1.8 -/-
Na v1.9 -/-

Supplementary table S1 – Primers used for quantitative real-time PCR

Gene	Primer direction	Primer Sequence	Product Size
Murine HPRT	FW	ACAGGCCAGACTTTGTTGGAT	150 bp
	RV	ACTTGCGCTCATCTTAGGCT	
Murine TRPV4	FW	GGACCCTGGCAAGAGTGAAATC	301 bp
	RV	CACCGGACAAATGCCTAAATGT	
MurineTRPA1	FW	TCCAAATTTTCCAACAGAAAAGGAA	147 bp
	RV	TCGCTATTGCTCCACATTGC	
Murine TRPV1	FW	AGCTACTACAAGGGCCAGAC	120 bp
	RV	GAAGAAGTCCCCGTTAGCAG	
Murine Nav1.7	FW	GTGATTGGGAACCTTGTGGT	114 bp
	RV	AATCTGGAGGTTGTTTGCGT	
Murine Nav1.8	FW	GTGTGCATGACCCGAACTGAT	101 bp
	RV	CAAAACCCTCTTGCCAGTATCT	
Murine Nav1.9	FW	CAGTCGTCCTAGCAGTAACA	151 bp
	RV	TCCGATGACAATGAAGTCCA	
